# Suppression of Natural Killer Cells by Sorafenib Contributes to Prometastatic Effects in Hepatocellular Carcinoma

**DOI:** 10.1371/journal.pone.0055945

**Published:** 2013-02-08

**Authors:** Qiang-Bo Zhang, Hui-Chuan Sun, Ke-Zhi Zhang, Qing-An Jia, Yang Bu, Miao Wang, Zong-Tao Chai, Quan-Bao Zhang, Wen-Quan Wang, Ling-Qun Kong, Xiao-dong Zhu, Lu Lu, Wei-Zhong Wu, Lu Wang, Zhao-You Tang

**Affiliations:** Liver Cancer Institute and Zhongshan Hospital, Fudan University, Key Laboratory for Carcinogenesis and Cancer Invasion, The Chinese Ministry of Education, Shanghai, People’s Republic of China; Istituto Superiore di Sanità, Italy

## Abstract

Sorafenib, a multi-tyrosine kinase inhibitor, is a standard treatment for advanced hepatocellular carcinoma (HCC). The present study was undertaken to determine whether the growth and metastasis of HCC were influenced in mice receiving sorafenib prior to implantation with tumors, and to investigate the *in-vivo* and *in-vitro* effect of sorafenib on natural killer (NK) cells. In sorafenib-pretreated BALB/c nu/nu mice and C57BL/6 mice, tumor growth was accelerated, mouse survival was decreased, and lung metastasis was increased. However, the depletion of NK1.1^+^ cells in C57BL/6 mice eliminated sorafenib-mediated pro-metastatic effects. Sorafenib significantly reduced the number of NK cells and inhibited reactivity of NK cells against tumor cells, in both tumor-bearing and tumor-free C57BL/6 mice. Sorafenib down-regulated the stimulatory receptor CD69 in NK cells of tumor-bearing mice, but not in tumor-free mice, and inhibited proliferation of NK92-MI cells, which is associated with the blocking of the PI3K/AKT pathway, and inhibited cytotoxicity of NK cells in response to tumor targets, which was due to impaired ERK phosphorylation. These results suggest immunotherapeutic approaches activating NK cells may enhance the therapeutic efficacy of sorafenib in HCC patients.

## Introduction

Hepatocellular carcinoma (HCC) is the fifth-most prevalent malignant tumor in men worldwide and the second-most frequent cause of cancer death [1]. Although surgical resection and liver transplantation are the main modalities of curative treatment for HCC, most patients present late stages of the disease, when curative treatment is not feasible and outcomes are likely to be poor [2]. Although nonsurgical treatments for HCC are available, such as radiofrequency ablation and transcatheter arterial chemoembolisation, the overall survival rate is not satisfactory [3]. In recent years, small-molecule kinase inhibitors, especially those targeting vascular endothelial growth factor (VEGF) and its receptor (VEGFR), have demonstrated a survival benefit. Sorafenib was the first molecularly targeted agent approved for treating advanced HCC [4]–[5]. Although the SHARP and ORIENTAL trials demonstrated sorafenib’s survival benefit, its efficacy is only moderate, because the response rate is actually quite low (2%–3%) and the survival benefit is only a few months [6]–[7]. The incidence of brain metastasis was found to be increased in HCC patients treated with drugs targeting VEGF or VEGFR [8]. It was also reported that sunitinib accelerated metastatic tumor growth and decreased overall survival in a mouse model; the authors suggested an effect on the formation of a prometastatic niche, but the underlying mechanism needs to be explored further [9]–[10]. It is conceivable that besides their effects on pathways in cancer cells, most targeted agents also have “off-target” effects on immune cells, including T cells, natural killer (NK) cells, monocytes and dendritic cells (DCs) [11]–[14]. However, little is known about the relationship between their prometastatic effect and the modulation of antitumor immunity.

NK cells, a major component of the innate immune system, can limit the growth and dissemination of several types of tumors [15]. Unlike T cells and B cells, NK cells can exert direct cellular cytotoxicity on tumor cells without prior sensitization and secrete immunostimulatory cytokines like interferon gamma (IFN-γ), which controls both local tumor growth and metastasis [16]. An epidemiologic survey showed that low NK cell activity is associated with increased cancer risk [17]. Many other independent studies revealed that NK cells have a role in the control of newly arising tumors in mice. Schreiber’s group demonstrated that frequencies of spontaneously arising tumors or tumors induced by methylcholanthrene were higher in mice deficient for key effector molecules of NK cells or the respective receptors [18]–[19]. Notably, some molecularly targeted agents have exhibited off-target effects on NK cells, in addition to direct effects on tumor cells. For example, imatinib can act on host DCs to promote NK cell activation, and rapamycin significantly inhibited proliferation and cytotoxicity of NK cells [20]–[21]. An in vitro study also demonstrated that pharmacological concentrations of sorafenib can affect the function of NK cells [12].

To elucidate the impact of sorafenib on host immunity, we investigated its effect on antitumor immunity, mainly T cells and NK cells. We found that tumor growth and metastasis increased and mouse survival decreased in a sorafenib-pretreated xenograft model; this could be attributable to a direct inhibitory effect of sorafenib on proliferation and activation of NK cells.

## Materials and Methods

### Cell Lines and Animals

NK92-MI, K562, Raji, human HCC cell line HepG_2_, and mouse cell line Hepa1-6, YAC-1 were obtained from the American Type Culture Collection. Human cell line HCC-LM_3_ was established at our institute. The stable red fluorescent protein (RFP)–expressing LM_3_-RFP and green fluorescent protein (GFP)–expressing Hepa1-6-GFP and HepG_2_-GFP cell lines, derived from LM_3_, Hepa1-6, and HepG_2_ cells, respectively, were kindly provided by Professor Wu WZ and were used in in vivo experiments [22].

Male BALB/c nu/nu mice and male C57BL/6 mice aged 4 to 6 weeks and weighing 20 g were obtained from the Shanghai Institute of Materia Medica, Chinese Academy of Science, and were maintained under specific pathogen-free conditions.

### Isolation of NK Cells

NK cells were isolated from peripheral blood of healthy donors or murine spleen by negative selection, using the NK Cell Isolation Kit and MACS columns (Miltenyi Biotec), and cultured with RPMI1640 medium supplemented with 10% fetal bovine serum (FBS). Experiments were performed when the purity of mouse NK1.1^+^ NK cells or human CD56^+^ NK cells was greater than 90%, as determined by flow cytometry. The study was approved by the research ethics committee of Zhongshan Hospital.

### Drugs and Treatment

Sorafenib (Bayer Healthcare, Inc) was suspended in vehicle solution that contained Cremophor (Sigma), 95% ethanol, and deionized water in a ratio of 1∶1:6 [23]. Pretreatment with vehicle or sustained sorafenib was initiated 2 weeks and was stopped 24 hours before the implantation of tumors in animal models. Recombinant human IL-2 (specific activity >10^7^units/mg) was purchased from Peprotech (USA). U0126 and PD98059 (inhibitors of both MEK1 and MEK2), LY294002 (inhibitor of phosphoinositide 3-kinase), and AG490 (inhibitor of Jak-2 protein tyrosine kinase) were purchased from Sigma and dissolved in dimethylsulfoxide (DMSO) for further experiments.

### Analysis by Flow Cytometry

Expression of cell surface antigens was measured by flow microfluorometry, as previously described in detail [24]. Briefly, spleen or PMBC cells were washed once with phosphate buffer solution (PBS) supplemented with 2% FBS and 0.05% sodium azide (2% FBS-PBS). The washed cells were incubated for 30 min at 4°C in 2% FBS-PBS with anti–human CD3; anti–human CD56 (Miltenyi Biotec, Germany); anti–human CD107a; anti–human NKG2D; anti–mouse CD3, CD4, CD8, NK1.1, and CD69 (BD Pharmingen, USA); or normal mouse serum, as a negative control. They were then washed twice with 2% FBS-PBS. Their fluorescence intensity was measured with a FACScan (BD Biosciences).

### Proliferation Assay

NK-92MI cells (10,000/well) were cultured in 96-well plates in the presence or absence of sorafenib. Proliferation was measured at different times using a Cell Counting Kit (Dojin Laboratories, Kumamoto, Japan), as previously described [25].

### Degranulation Assay

The purified human CD56^+^ NK cells from healthy donors were cocultured with K562 cells at an E:T ratio of 3∶1 for 2 h. Subsequently, cells were stained with anti–CD107a-FITC or isotype control. Analysis was performed on a FACScan.

### Cytotoxicity Assay

The isolated human CD56^+^ NK cells or isolated murine NK1.1^+^ cells were cultured in RPMI 1640 medium with various concentrations of sorafenib or inhibitors. Cytotoxicity was determined by lactate dehydrogenase (LDH) release assays, as previously described [26]. The K562 and YAC-1 cells were used as targets to evaluate NK activity. In all experiments, spontaneous release was less than 15% of maximum release. In brief, these effector and target cells were plated at an appropriate E:T ratio in 96-well, round-bottomed plates. After incubation for 4 h, the LDH in the medium was measured with the nonradioactive Cytotoxicity Detection Kit^PLUS^ (LDH) (Roche, Mannheim, Germany). Determinations were carried out in triplicate. The percentage of specific cytolysis was calculated from the release of LDH from test samples and control samples, as follows:
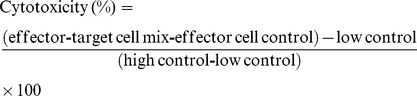



### Determination of IFN-γ

IFN-γ concentrations in cell culture supernatants were analyzed by ELISA, with the Quantikine ELISA kits (R&D Systems). All analyses were carried out in duplicate.

### Western Blot Analysis

Western blot analysis with whole-cell extracts was performed essentially as previously described elsewhere [24]. Primary antibodies included anti-pERK1/2, anti-pAKT (Cell Signaling Technology; Danvers, MA, USA), anti-ERK1/2, and anti-AKT (Abcam; Cambridge, MA, USA).

### HCC Animal Models

For the subcutaneous tumors, (3×10^6^ cells/200 µL PBS/mouse) were injected subcutaneously into the flanks of BALB/c nu/nu mice. The formation and size of the tumors were monitored every 4–5 days until Day 35. Tumor area was calculated as *A* = *a*×*b*, where *a* is the longest diameter and *b* is the shortest diameter of the tumor.

For the orthotopic HCC tumors in animal models, tumor cells were subcutaneously inoculated into the right flanks of 4-week-old BALB/c nu/nu or C57BL/6 male mice. After 3–4 weeks, non-necrotic tumor tissue was cut into 1 mm×1 mm pieces and orthotopically implanted into the liver.

For experimental lung metastases of cancer cells, viable tumor cells suspended in 200 µL PBS were injected into the lateral tail vein of mice [27]. In some experiments, C57BL/6 mice received intraperitoneal injections with neutralizing anti-NK1.1 mAb (300 µg of PK136 mAb/mouse) on days −4, −2, 0, 5, and 10 in C57BL/6 mice before tumor inoculation, as described by other authors [19].

Five weeks after implantation of tumors, the mice were sacrificed, and fluorescent protein–positive (GFP^+^ or RFP^+^) metastatic foci were imaged (stereomicroscope: Leica MZ6; illumination: Leica L5FL; C-mount: 0.63/1.25; charge-coupled device: DFC 300FX). Integrated optical density (IOD) was quantified by Image-Pro Plus software (Media Cybernetics; Bethesda, MD) [22].

### Ethics Statement

The study was approved by the research ethics committee of Zhongshan Hospital and the Shanghai Medical Experimental Animal Care Commission. Peripheral blood used in the study was obtained with informed written consent from each healthy donor.

### Statistical Analysis

Continuous data were expressed as means ± standard errors. Comparisons were made with unpaired, 2-tailed Student *t* test, 1-way ANOVA, or Mann-Whitney *U* test. All statistical analyses were conducted with SPSS 16.0 (SPSS; Chicago, IL, USA).

## Results

### Sorafenib Pretreatment Accelerated Tumor Growth, Promoted Lung Metastasis, and Decreased Overall Survival in Nude Mice

**Figure 1:**
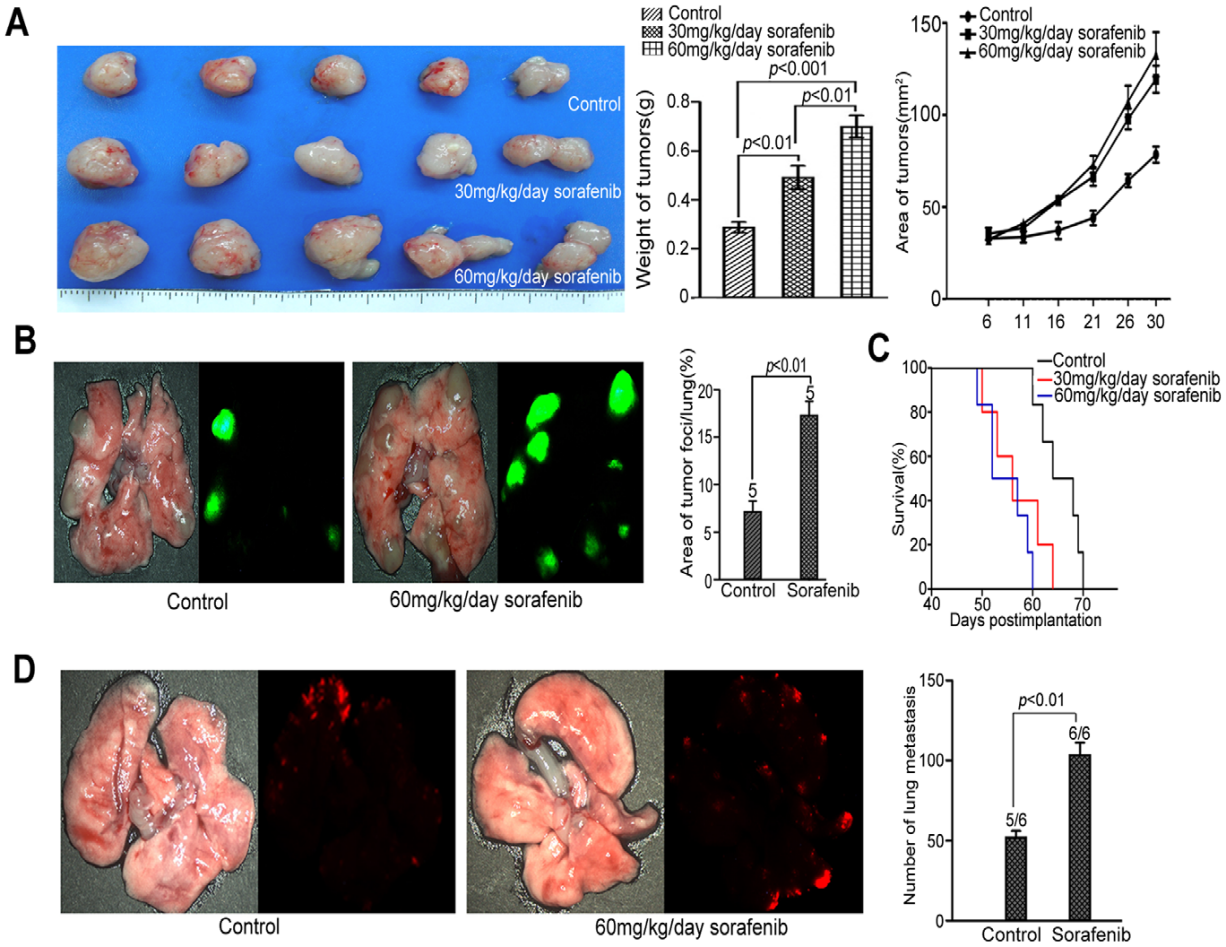
Sorafenib pretreatment accelerated tumor growth, promoted lung metastasis, and decreased overall survival in nude mice. (A) In the HepG_2_ subcutaneous tumors model, sorafenib pretreatment accelerated the growth of ectopic tumors in nude mice. Tumor weight was 0.31±0.05 g, 0.51±0.09 g, and 0.73±0.09 g in the vehicle, 30 mg·kg^−1^·day^−1^ sorafenib, and 60 mg·kg^−1^·day^−1^ sorafenib groups, respectively (n = 5 for each group) (*p*<0.01, right panel). (B) In the HepG_2_-GFP experimental lung metastases model, sorafenib pretreatment enhanced the formation of lung metastatic foci, which was 7.19±2.16% in the vehicle group and 17.32±2.92% in the sorafenib group (n = 5 for each group) (*p*<0.01, right panel). (C) In the LM_3_-RFP orthotopic model of nude mice, sorafenib pretreatment increased the number of lung-metastatic foci, compared to the vehicle group (n = 6 for each group) (105.0±19.80 versus 59.7±16.8 per lung, *p*<0.01, right panel), but did not increase the rate of lung metastasis, which was 6 of 6 in the sorafenib group and 5 of 6 in controls. (D) Sorafenib significantly reduced median survival compared to vehicle in the nude mice inoculated HepG_2_ cells. Mice pretreated with 30 mg·kg^−1^·day^−1^ and 60 mg·kg^−1^·day^−1^ sorafenib survived 52 and 56 days, respectively, compared to 64 days for the controls (*p*<0.01).

To evaluate the effect of immunosuppressive properties of sorafenib on tumorigenicity and tumor growth, we inoculated LM_3_ cells into the flanks of sorafenib-pretreated (30 mg·kg^−1^·day^−1^ and 60 mg·kg^−1^·day^−1^, 2 weeks) and vehicle-pretreated BALB/c nu/nu mice and measured tumor size every 4–5 days until Day 35. We found sorafenib pretreatment significantly promoted tumor growth, compared to the vehicle-pretreated mice **(** **Fig. 1A** **).**

Next, we examined the metastatic potential of tumor cells in sorafenib-pretreated mice. HepG_2_-GFP cells (1×10^6^) were inoculated into the tail veins of sorafenib-pretreated (60 mg·kg·^−1^day^−1^, 2 weeks) and vehicle-pretreated nude mice, and the mice were sacrificed on Day 42. More lung metastatic foci were found in the sorafenib-pretreated mice **(** **Fig. 1B** **)**, and survival time was significantly shorter in sorafenib-pretreated mice, compared with vehicle-pretreated mice **(** **Fig. 1C** **).**

We also established LM_3_-RFP (high potential to form lung metastasis) orthotopic HCC tumor model in sorafenib-pretreated (60 mg·kg·^−1^day^−1^, 2 weeks) and vehicle-pretreated nude mice. The mice were sacrificed 5 weeks after tumor implantation. As shown in **Fig. 1D** **,** lung metastasis was significantly increased by sorafenib pretreatment in the LM_3_-RFP model, although the difference in tumor volume was not statistically significant **(Fig. S1).**

### Sorafenib Pretreatment Promoted Lung Metastasis in Immunocompetent C57BL/6 Mice but not NK-cell-depleted C57BL/6 Mice

To verify the above findings in immunocompetent mice, we established experimental lung metastasis in C57BL/6 mice with mouse HCC cell line Hepa1-6-GFP. Again, enhanced lung metastasis was observed in sorafenib-pretreated (60 mg·kg·^−1^day^−1^) mice but not in vehicle-pretreated C57BL/6 mice.

**Figure 2:**
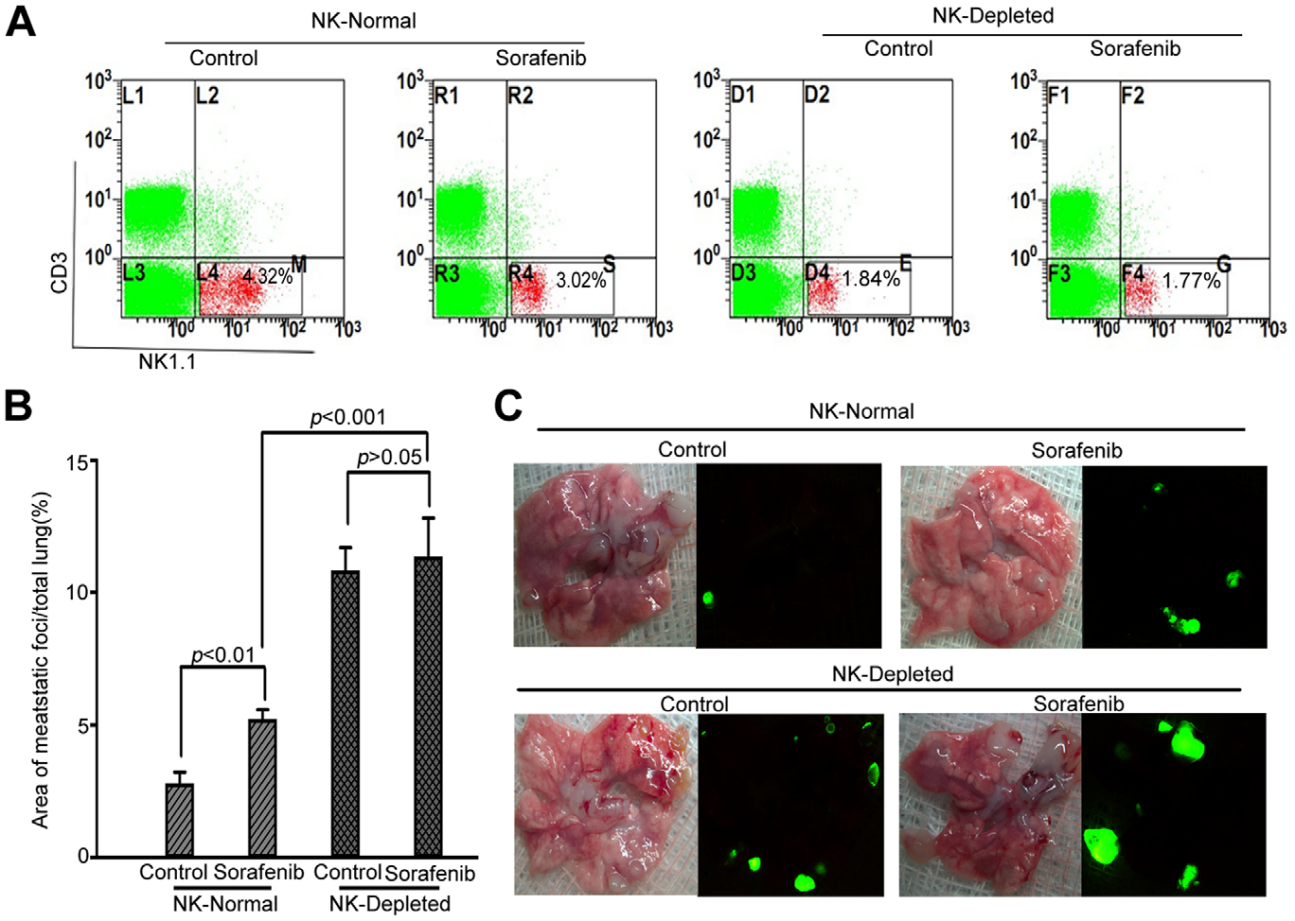
Sorafenib pretreatment promoted lung metastasis only in mice with intact NK cells. (A) At day 7, the ratio of NK cells in the spleens was determined by flow cytometry. (B) The metastatic index was measured by the ratio of the area of GFP-positive lung metastatic foci to total lung area. There was no significant difference between vehicle and sorafenib in NK cell–depleted groups (n = 5 for each group).

Based on the above findings in nude mice and C57BL/6 mice, we hypothesize that NK cells were suppressed by sorafenib pretreatment, resulting in enhanced tumor growth and metastasis. To directly analyze the contribution of NK cells to the prometastatic effect of sorafenib pretreatment, the same number of Hepa1-6-GFP cells were injected into the tail veins of NK-cell-depleted C57BL/6 mice pretreated with sorafenib or vehicle **(** **Fig. 2A** **).** Notably, depletion of NK cells significantly promoted the formation of lung metastatic foci compared to the NK-normal C57BL/6 mice, but it eliminated the prometastatic effect of sorafenib pretreatment in NK-depleted C57BL/6 mice **(** **Fig. 2B, 2C** **).** We concluded that NK cells may play a critical role in the prometastatic effect of sorafenib.

### Sorafenib Reduced the Number of NK Cells in a Dose-dependent Manner

**Figure 3:**
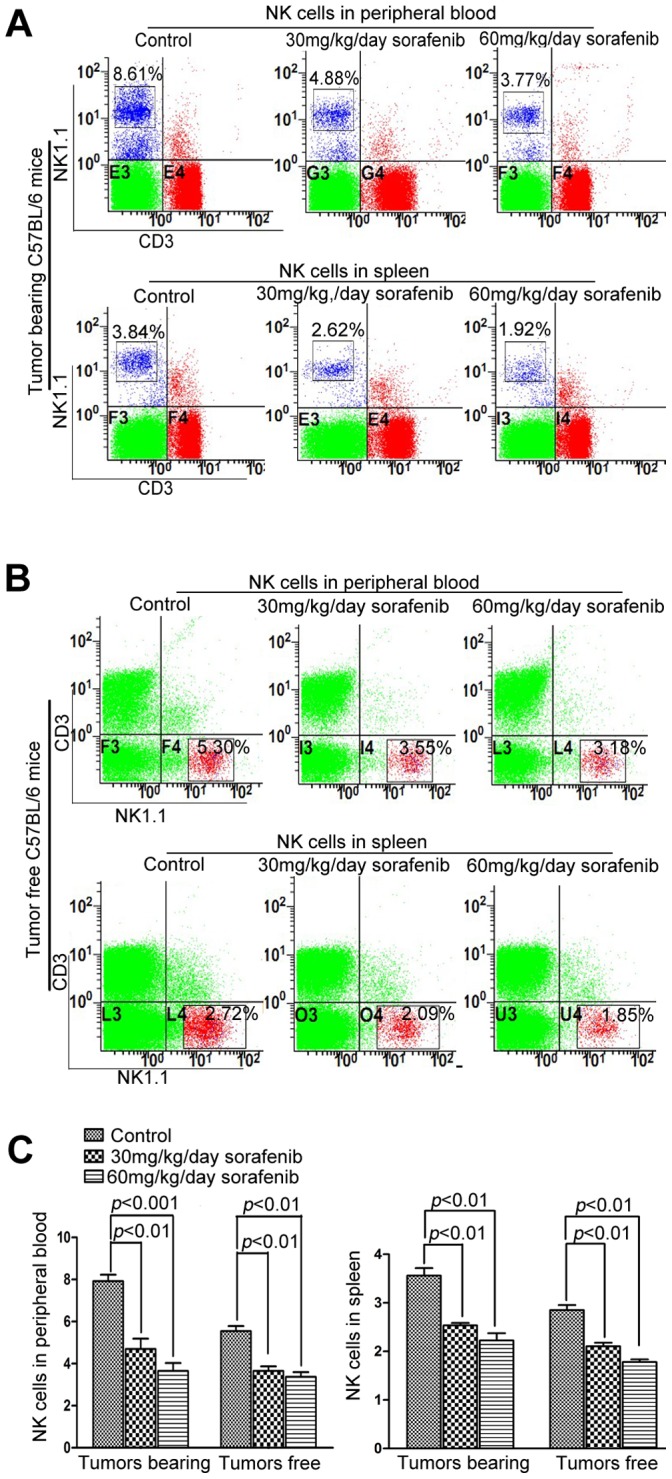
Sorafenib reduced the ratio of NK cells dose-dependently, in both tumor-free and tumor-bearing C57BL/6 mice. Sorafenib treatment was started one week after tumor implantation and continued for two weeks. Then, NK cells were harvested for further measurement. (A, C) The ratio of NK cells was 7.92±0.61% in the controls and 4.70±0.98%, and 3.66±0.74% in the 30 mg·kg^−1^·day^−1^ sorafenib and 60 mg·kg^−1^·day^−1^ sorafenib groups (n = 5 for each group), respectively (*p*<0.001). For tumor-bearing mice, the ratio was 3.56±0.31% in controls and 2.54±0.99% and 2.23±0.30% in the 30 mg·kg^−1^·day^−1^ sorafenib and 60 mg·kg^−1^·day^−1^ sorafenib groups, respectively (*p*<0.01). (B, C) Sorafenib also reduced the ratio of NK cells dose-dependently in the peripheral blood and spleens of tumor-free mice (n = 5 for each group): 2.85±0.21% in controls and 2.11±0.15% and 1.78±0.11% in the 30 mg·kg^−1^·day^−1^ and 60 mg·kg^−1^·day^−1^ groups, respectively, in the spleens (*p*<0.001, right panel); and 5.54±0.49% in the controls and 3.66±0.43% and 3.37±0.45% in the 30 mg·kg^−1^·day^−1^ and 60 mg·kg^−1^·day^−1^ groups, respectively, in peripheral blood.

After treating the mice for 2 weeks with 2 different doses of sorafenib, we examined the number of T cells and NK cells in the spleen and peripheral blood. As shown in **Fig. 3A and 3C** **,** in orthotopic Hepa1-6 tumor–bearing C57BL/6 mice, sorafenib decreased the number of NK cells (CD3^−^ and NK1.1^+^) in a dose-dependent manner, relative to the vehicle-pretreated mice. Although the total number of NK cells was lower in tumor-free mice than in tumor-bearing mice, sorafenib decreased the number of NK cells in both tumor-bearing and tumor-free mice **(** **Fig. 3B, C** **).** However, the changes of CD4^+^ T cells and CD8^+^ T cells in tumor-bearing mice were not significant **(Fig. S2).** Therefore, we concluded that sorafenib reduced the number of NK cells but not T cells in immunocompetent mice.

### Sorafenib Affected the Expression of CD69 and Cytotoxicity of NK Cells in Mice

**Figure 4:**
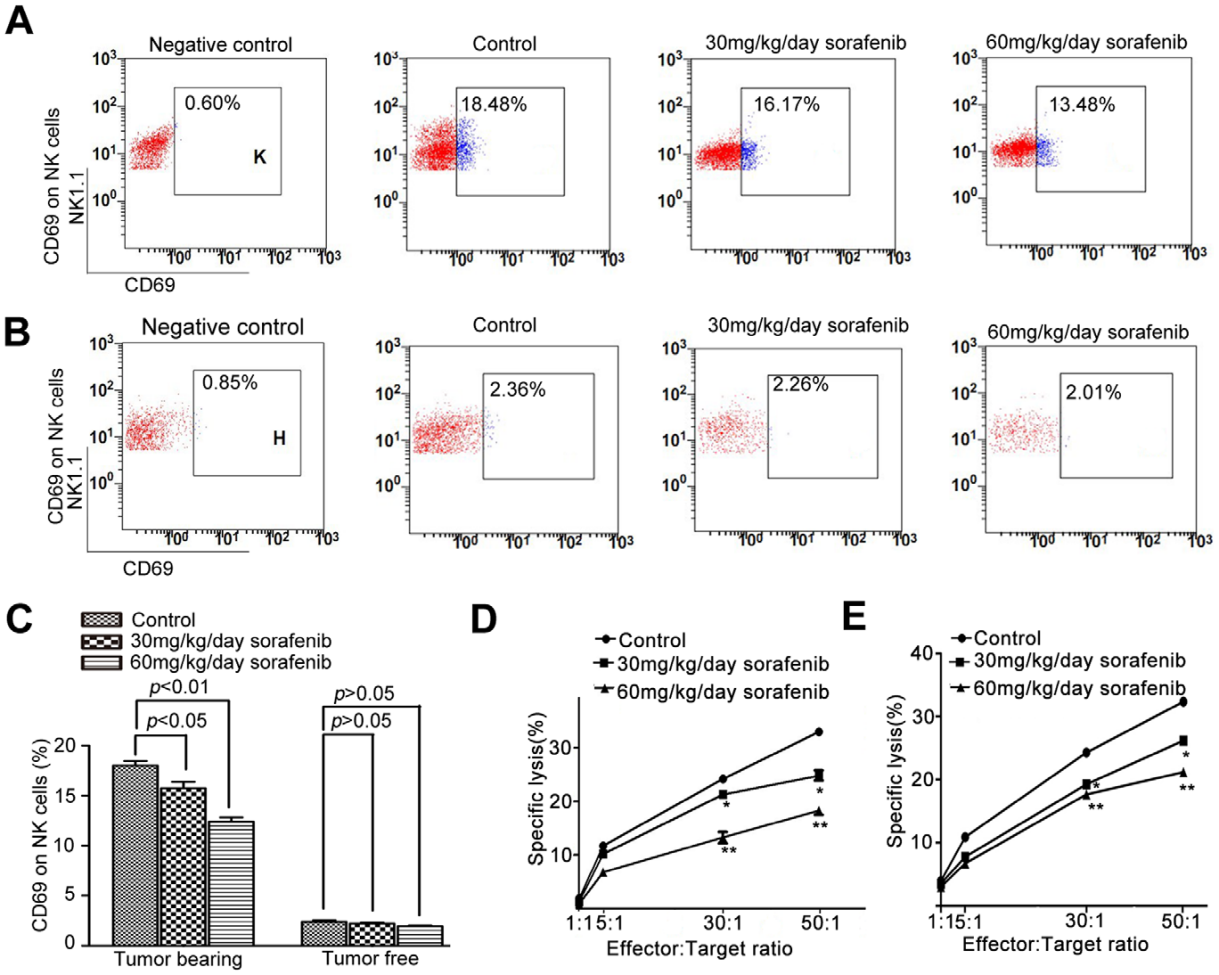
Sorafenib inhibited the activation and cytotoxicity reaction of NK cells in vivo. (A, C) The ratio of CD69^+^ NK cells was 18.0±0.91% in controls, and 15.75±1.22% and 12.41±0.85% in the 30 and 60 mg·kg^−1^·day^−1^ groups, respectively (*p*<0.05) (n = 5 for each group). (B, C) NK cells were not activated in tumor-free mice, and sorafenib did not reduce the expression of CD69 (*p*>0.05). (D, E) Sorafenib substantially inhibited the cytolytic activity of isolated NK cells in response to YAC-1 cells from both tumor-bearing mice and blank mice (n = 5 for each group). *, 30 mg·kg^−1^·day^−1^ sorafenib group compared with control group; **, 60 mg·kg^−1^·day^−1^ sorafenib group compared with control group; *,**,*P*<0.05.

We detected CD69 expression, a marker of activated NK cells, to study the function of NK cells [28]. The basal level of CD69 in NK cells from tumor-bearing C57BL/6 mice was greater than that of NK cells from tumor-free mice **(** **Fig. 4A–C** **).** CD69 expression was downregulated by sorafenib (30 mg·kg·^−1^day^−1^ and 60 mg·kg^−1^·day^−1^, 2 weeks) in tumor-bearing C57BL/6 mice but not in tumor-free mice.

To study whether sorafenib affects the cytotoxic activity of NK cells against tumor cells, we examined the effect of sorafenib on tumor-cell (YAC-1) lysis by NK cells, in both tumor-bearing and tumor-free mice. We isolated NK cells from sorafenib-pretreated and vehicle-pretreated C57BL/6 mice. Pretreatment with sorafenib (30 mg·kg^−1^·day^−1^ and 60 mg·kg^−1^·day^−1^, 2 weeks) caused a substantial reduction of NK-mediated cytotoxicity against YAC-1 cells in both tumor-bearing and tumor-free mice **(** **Fig. 4D, E** **).**

### Sorafenib Inhibited the Proliferation of NK Cells, Mainly by Blocking the PI3K/AKT Pathway

**Figure 5:**
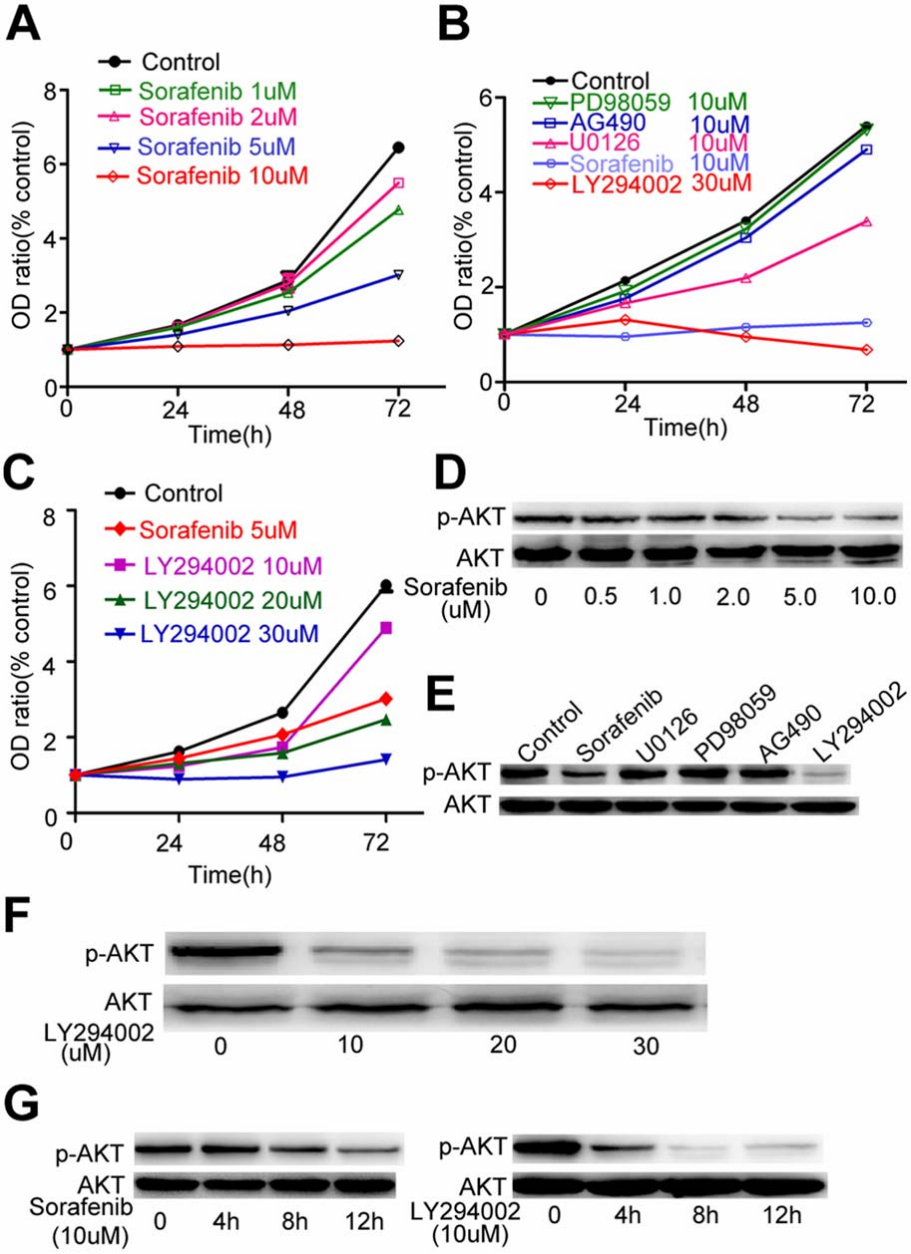
Sorafenib inhibited proliferation of NK-92MI cells mainly by blocking the PI3K/AKT pathway. (A) Sorafenib inhibited proliferation of NK-92MI cells dose-dependently. (B) Addition of different specific inhibitors had different effects on the proliferation of NK-92MI cells at the indicated time. (C) Different concentrations of LY294002 had an inhibitory effect on proliferation of NK-92MI cells at the indicated time. (D, E) Cells were treated with different concentrations of Sorafenib and LY294002 for 6 h. (F) Cells were treated with different inhibitors (10 µM) for 6 h. (G) Cells were treated with sorafenib (10 µM) and LY294002 (10 µM) for different lengths of time.

We used human NK cell line NK-92MI because it is derived from peripheral blood mononuclear cells from a patient with rapidly progressive non-Hodgkin’s lymphoma and has been used to study the function and signaling pathways of NK cells[29]–[30]. The rate of inhibition of NK-92MI cell proliferation depended on the concentration of sorafenib: more than 60% at 5 µM for 72 h, and almost 100% at 10 µM **(** **Fig. 5A** **).** We studied 3 signal transduction pathways, Raf/MEK/ERK, PI3K/AKT, and JAK/STAT3, to determine which was affected by sorafenib. We found that 30 µM PI3K-specific inhibitor LY294002 inhibited growth in a similar fashion as 10 µM sorafenib **(** **Fig. 5B** **),** indicating that sorafenib may affect the PI3K/AKT pathway in NK92-MI. Like sorafenib, LY294002 (10 µM, 20 µM, and 30 µM) had a dose-dependent effect on NK-92MI growth **(** **Fig. 5C** **).** Next, Western blot showed that the amount of phosphorylated Akt on Serine 473 is decreased by sorafenib after 12-h exposure **(** **Fig. 5D** **).** Akt phosphorylation was inhibited in a dose- and time-dependent manner by sorafenib or LY294002 **(** **Fig. 5E–G** **).** Thus, because phosphorylation of serine 473 of Akt was direct evidence of PI 3-kinase activation, these results suggest that the inhibition of PI 3-kinase is critical to the inhibitory effect of sorafenib on NK-92MI. Therefore, although treatment with AG490 and PD98059 did not affect cell growth**,** it is less likely that sorafenib inhibited proliferation of NK92-MI through JAK/STAT3 and Raf/MEK/ERK pathways.

### Sorafenib Impaired the Reactivity of Human NK Cells in Response of K562 Cells in vitro

**Figure 6:**
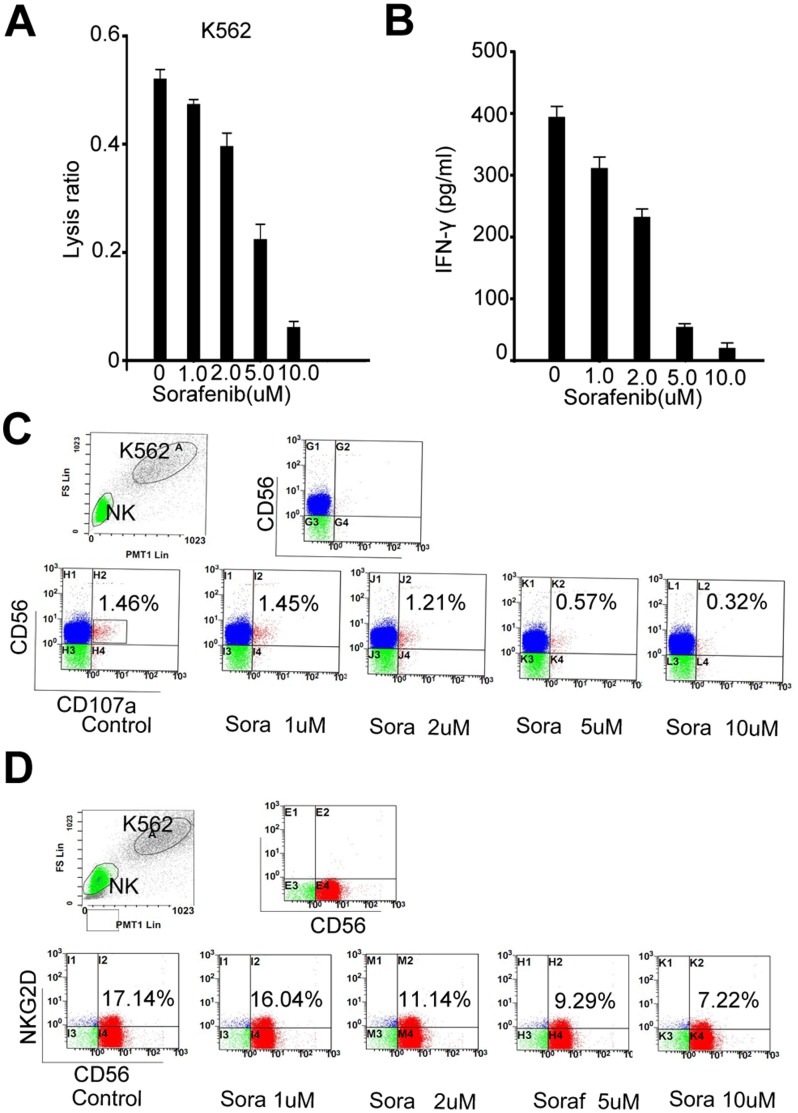
Sorafenib impaired the reactivity of isolated human NK cells in vitro. (A) Sorafenib reduced the lysis ratio of NK cells in response to K562 cells (E:T = 30∶1) in a dose-dependent manner. (B) Sorafenib decreased the production of IFN-γ in NK cells when cocultured with K562 at the ratio of 3∶1 for 12 h. (C) Sorafenib reduced the expression of CD107a in NK cells mixed with K562 cells at the ratio of 3∶1 for 2 h. Representative flow cytometry dot plots were showed. (D) The expression of NKG2D by NK cells was also reduced when they were mixed with K562 cells at the ratio of 3∶1 for 8 h. Representative flow cytometry dot plots were showed.

We investigated whether sorafenib affects freshly isolated NK cells from a healthy donor. We mixed freshly isolated NK cells with K562 (E:T ratio 30∶1) for 4 h in the presence of different concentrations of sorafenib and evaluated lysis activity by LDH assay [26]. Sorafenib treatment caused substantial reduction of NK-mediated cytotoxicity against K562 cells **(** **Fig. 6A, S** **3).** In addition, sorafenib also dose-dependently impaired IFN-γ production in NK cells cocultured with K562 cells **(** **Fig. 6B** **)**. Furthermore, we performed degranulation assays in cocultures of NK and K562 cells. Presence of K562 cells induced expression of CD107a in NK cells, a marker of activated NK cells [31], and sorafenib significantly reduced its expression in a dose-dependent manner **(** **Fig. 6C** **).** In line with the results obtained in analysis of cytotoxicity and degranulation, sorafenib treatment also drastically altered the expression of stimulatory receptor NKG2D **(** **Fig. 6D** **).** The results indicate that sorafenib has a direct impact on NK cells, and this is a basis of immunosuppression.

### Sorafenib Inhibits Raf/MEK/ERK Signaling Involved in NK Activation

**Figure 7:**
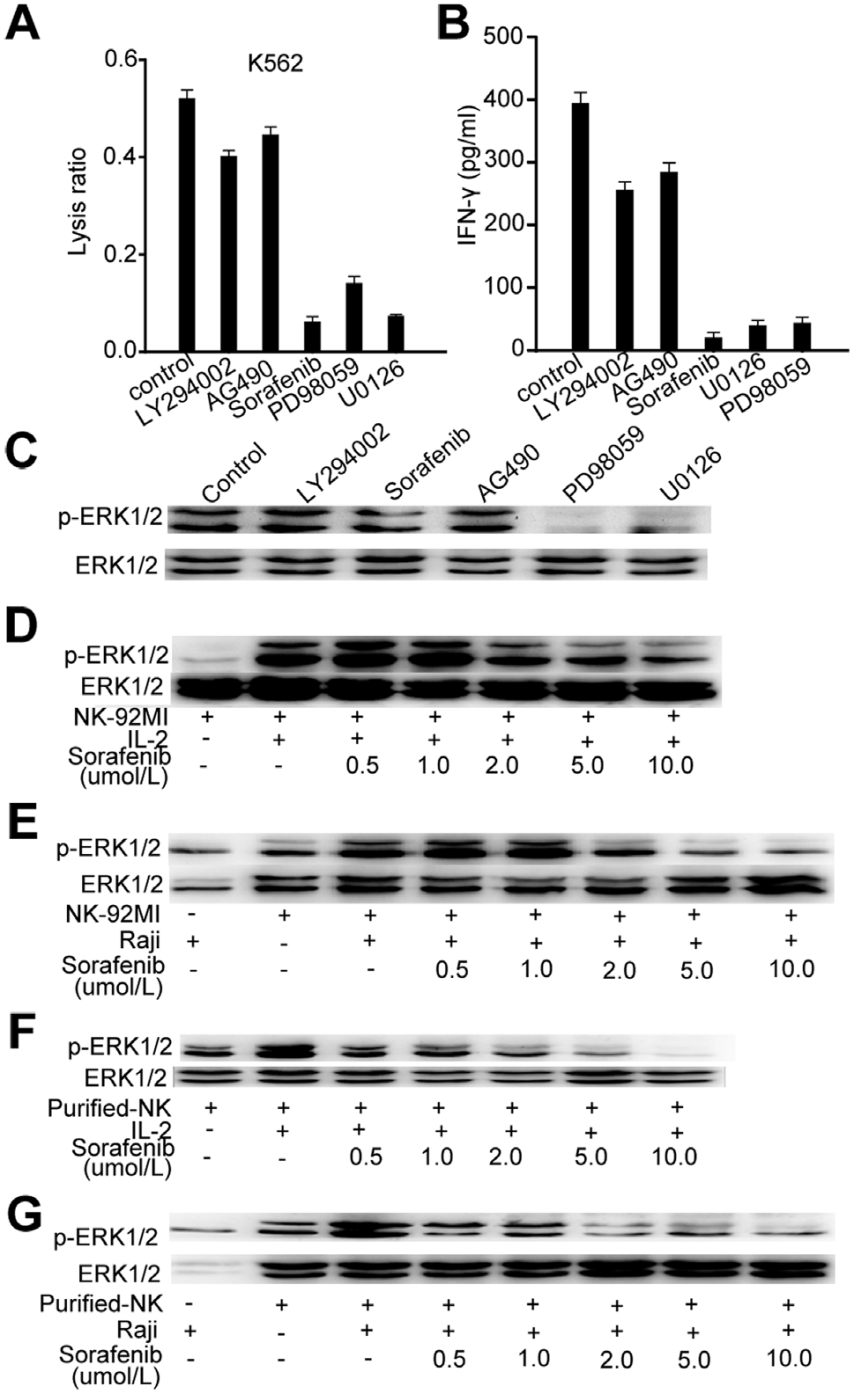
Sorafenib inhibited Raf/MEK/ERK signaling involved in NK activation. (A) NK cells were cultured with K562 cells (E:T = 30∶1) in the presence of sorafenib (10 µM) and the indicated specific inhibitors (10 µM). (B) NK cells were cultured with K562 tumor cells in the presence of sorafenib (10 µM) and the indicated specific inhibitors (10 µM). After 24 h, IFN-γ levels in cell culture supernatants were determined by ELISA. (C) Isolated human NK cells were treated with sorafenib and the indicated specific inhibitors for 30 min and exposed to IL-2 for 10 min. Subsequently, cell lysates were analyzed for phosphorylated ERK (pERK1/2) and whole (panERK) ERK1/2 protein. (D, E) NK92-MI cells were treated with different concentrations of sorafenib for 30 min and exposed to IL-2 for 10 min or fixed Raji cells for 15 min. Subsequently, cell lysates were analyzed as described in C. (F, G) Isolated human NK cells were treated with different concentrations of sorafenib for 30 min and exposed to IL-2 for 10 min or fixed Raji cells for 15 min.

To determine which signal transduction pathway was affected by sorafenib in the freshly isolated NK cells, we added specific signaling inhibitors for Raf/MEK/ERK, PI3K/AKT, or JAK/STAT3 to the NK and K562 coculture. Addition of the specific MEK inhibitors PD98059 and U0126, 20 µM, was followed by cytotoxicity and IFN-γ production similar to that observed with 10 µM sorafenib **(** **Fig. 7A, B** **),** suggesting inhibition of ERK1/2 pathway may mediate the impairment of NK cell reactivity by sorafenib.

Addition of specific MEK inhibitors PD98059 and U0126 almost eliminated IL-2–induced ERK1/2 phosphorylation of NK-92MI cells **(** **Fig. 7C** **).** Presence of Raji cells and IL-2 induced substantial ERK1/2 phosphorylation in NK-92MI cells, while sorafenib moderately reduced detectable levels of ERK1/2 phosphorylation at the low concentration of 2 µM and almost eliminated ERK1/2 phosphorylation at 10 µM **(** **Fig. 7D,E** **).** Moreover, we used isolated NK cells from a healthy donor to analyze ERK1/2 phosphorylation in the presence of different concentrations of sorafenib. Notably, a much lower concentration (0.5 µM) of sorafenib substantially reduced the level of ERK1/2 phosphorylation induced by Raji cells and IL-2 in isolated NK cells at **(** **Fig. 7F,G** **);** this may be because isolated NK cells are more sensitive to sorafenib than NK-92MI cells. Together, these data demonstrate that sorafenib impaired the reactivity of NK cells mainly by inhibiting the ERK signaling pathway.

## Discussion

The present studies demonstrated that the off-target effects of sorafenib on host immunity may promote tumor growth and metastasis and shorten host survival in a unique model of sorafenib pretreatment. Other authors have reported similar observations [9]–[10], however we also found that the immunosuppressive property of sorafenib was mediated by suppression of NK cells; the relevance of suppression of T cells is less likely. Furthermore, sorafenib inhibits NK cells by directly affecting their proliferation and function. This mechanism is mediated by the blocking of AKT and ERK phosphorylation.

Because the protumor effect of sorafenib was similar in nude mice and the C57BL/6 model, we think NK cells may be more important than T cells in mediating its off-target effect. We found sorafenib pretreatment could accelerate the formation of metastatic foci only in mice with intact NK cells; this hints that the inhibition of NK cells might mediate off-target effects of sorafenib. The in vivo analyses relied on NK1.1-mediated depletion of NK cells in mice [32]. However, NK cell depletion with antibodies to NK1.1 may also affect populations of other immune cells, such as invariant natural killer T (NKT) cells and other NK1.1^+^ T-cell populations. We concluded that NK cells may play a critical role in sorafenib’s pro-tumor effect.

Several preclinical studies reported immunomodulatory effects of sorafenib, but the results are not conclusive. In cell culture, concentrations of sorafenib normally achieved in patients led to irreversible inhibition of proliferation of human peripheral T cells, whereas higher concentrations resulted in apoptosis [13]. Another report demonstrated that sorafenib can enhance antitumor immunity in a murine liver cancer model `by decreasing the suppressive immune cell populations (regulatory T cells and myeloid-derived suppressor cells) [33]. Although an in vitro study provided evidence that sorafenib inhibited the cytotoxicity of NK cells [12], little is known about the effects of sorafenib on NK cell proliferation and activation in vivo, and on tumor growth and dissemination [11]. We found that the proliferation and cytotoxicity of in vivo NK cells were inhibited by sorafenib at 5 µM, which is within the range of serum concentrations of patients treated with the standard dosage of 400 mg, twice daily [34]. The present study demonstrated a potential risk of immunosuppression in patients treated with this drug and highlighted the importance of testing additional drugs in combination as a possible approach to eliminate this effect.

Another important finding is that sorafenib (60 mg·kg^−1^·day^−1^) did not significantly change the number of CD4^+^ and CD8^+^ T cells in tumor-bearing mice. Zhao et al reported that sorafenib reduced the percentage of dividing CD4^+^ T cells in lymph nodes [13]. It should be noted that the dosage of sorafenib in Zhao’s study, 80 mg·kg^−1^·day^−1^, was higher than the dosage in our study; and T cells used in Zhao’s study were stimulated by specific OVA_323–339_ T antigens and LCK kinase phosphorylation was activated. That could explain the discrepancy between Zhao’s results and our own.

ERK1/2 activation has been demonstrated to be crucial for NK cell–mediated cytotoxicity in a plethora of models [35]–[36], while PI 3-kinase is reported to mediate IL-2–induced NK cell growth and survival [37]. The sorafenib mechanism relies on blocking Raf/MEK/ERK, PI3K/AKT, and Jak/STAT3 pathways in different models [38]–[40]. We found that the blocking of ERK1/2 phosphorylation was more relevant to the impairment of NK cell activation compared with other pathways, and AKT phosphorylation was associated with survival of NK92-MI cells. The effects of sorafenib on NK cell activation may be due to inhibition of signaling events earlier than 1 h and survival by the inhibition of signaling events later than 12 h. On the other hand, it should be noted that the proliferation of in vitro NK92-MI cells may be different from the proliferation of normal NK cells in vivo.

Because of the limitation of the study design, we did not observe other mechanisms that may be involved in a pro-tumor effect. For example, sorafenib may disturb immunity-related cytokines or other immunocytes, like dendritic cells.

Our findings suggest that sorafenib inhibited NK cells, both in number and function, rendering the host more susceptive to tumor growth and metastasis. Sorafenib could inhibit the proliferation of NK cells and impair their reactivity by affecting phosphorylation of AKT and ERK1/2, respectively. Therefore, immunotherapeutic approaches to activate NK cells may potentially compensate for the off-target effect of sorafenib in patients with HCC.

## Supporting Information

Figure S1 **In the LM_3_-RFP orthotopic model of nude mice, there was no difference in the volume of the orthotopic tumor between sorafenib-pretreatment group (60 mg·kg^−1^·day^−1^, 2 weeks) and the controls** ***(p>*** **0.05, right panel).**
(TIF)

Figure S2 **The number of CD4^+^T cells and CD8^+^ T cells didn’t changed obviously in the controls and sorafenib-treated tumor-bearing C57BL/6 mice.** The ratio of CD4^+^ T cells was 21.21±1.04% in the controls and 20.51±1.05% in the group treated with 60 mg·kg^−1^·day^−1^ sorafenib (*p* = 0.38, lower panel). The ratio of CD8^+^ T cells was 13.14±1.39% in the controls and 14.04±1.57% in the group treated with 60 mg·kg^−1^·day^−1^ sorafenib (*p* = 0.42, lower panel).
(TIF)

Figure S3 **Presence of different concentrations of sorafenib reduced the lysis ratio of NK cells in response to K562 cells (E: T = 30∶1).**
(TIF)
