# Supplementary figures and images for: Suppression of Natural Killer Cells by Sorafenib Contributes to Prometastatic Effects in Hepatocellular Carcinoma
Source: PLoS One. 2013 Feb 8;8(2):e55945. doi: 10.1371/journal.pone.0055945 (PMC3568028; doi:10.1371/journal.pone.0055945)

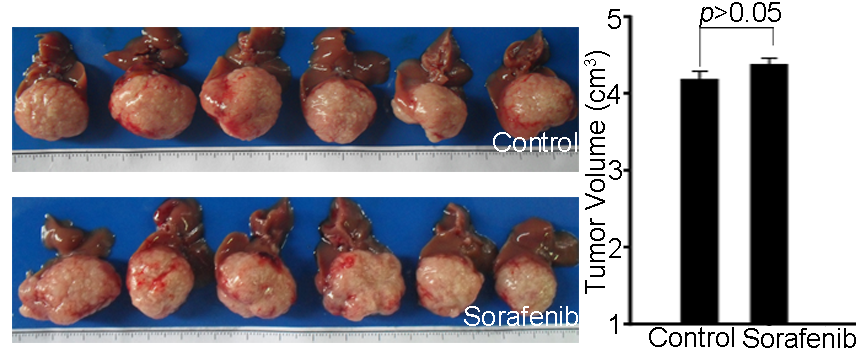

Supplement: Figure S1 — In the LM3-RFP orthotopic model of nude mice, there was no difference in the volume of the orthotopic tumor between sorafenib-pretreatment group (60 mg·kg−1·day−1, 2 weeks) and the controls (p> 0.05, right panel). (TIF) [file pone.0055945.s001.tif]

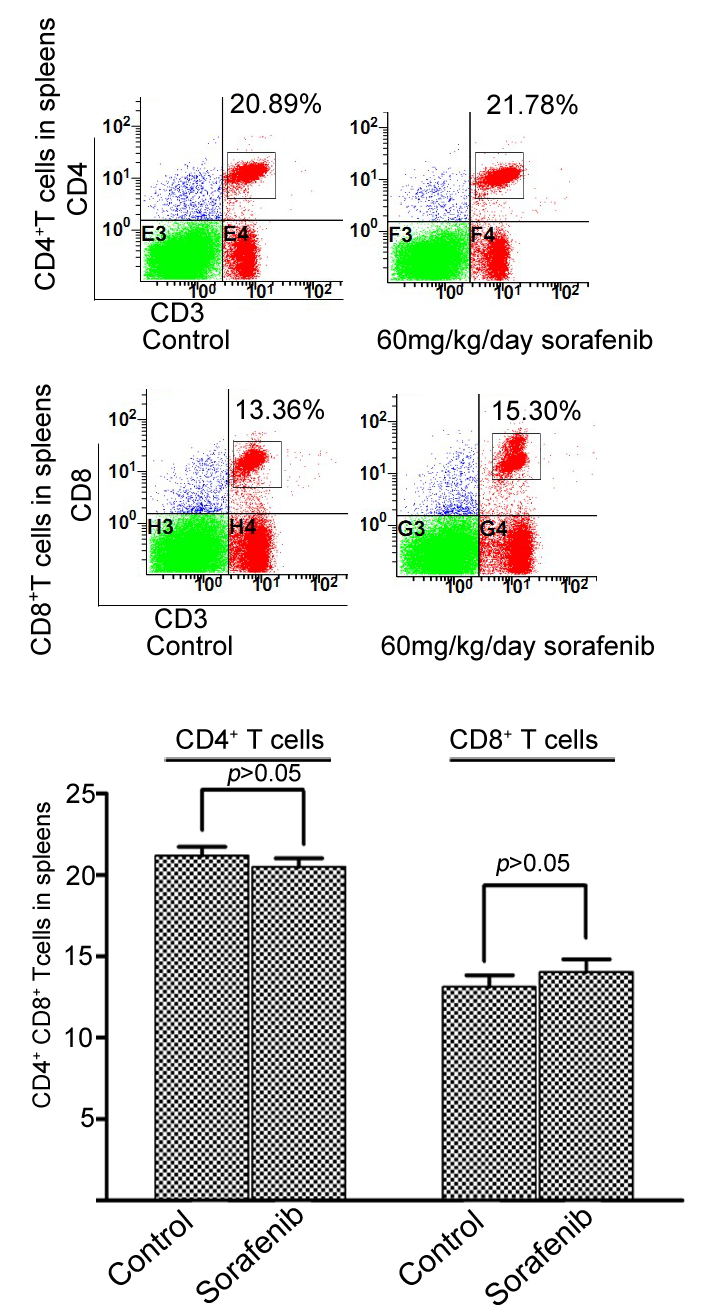

Supplement: Figure S2 — The number of CD4+T cells and CD8+ T cells didn’t changed obviously in the controls and sorafenib-treated tumor-bearing C57BL/6 mice. The ratio of CD4+ T cells was 21.21±1.04% in the controls and 20.51±1.05% in the group treated with 60 mg·kg−1·day−1 sorafenib (p = 0.38, lower panel). The ratio of CD8+ T cells was 13.14±1.39% in the controls and 14.04±1.57% in the group treated with 60 mg·kg−1·day−1 sorafenib (p = 0.42, lower panel). (TIF) [file pone.0055945.s002.tif]

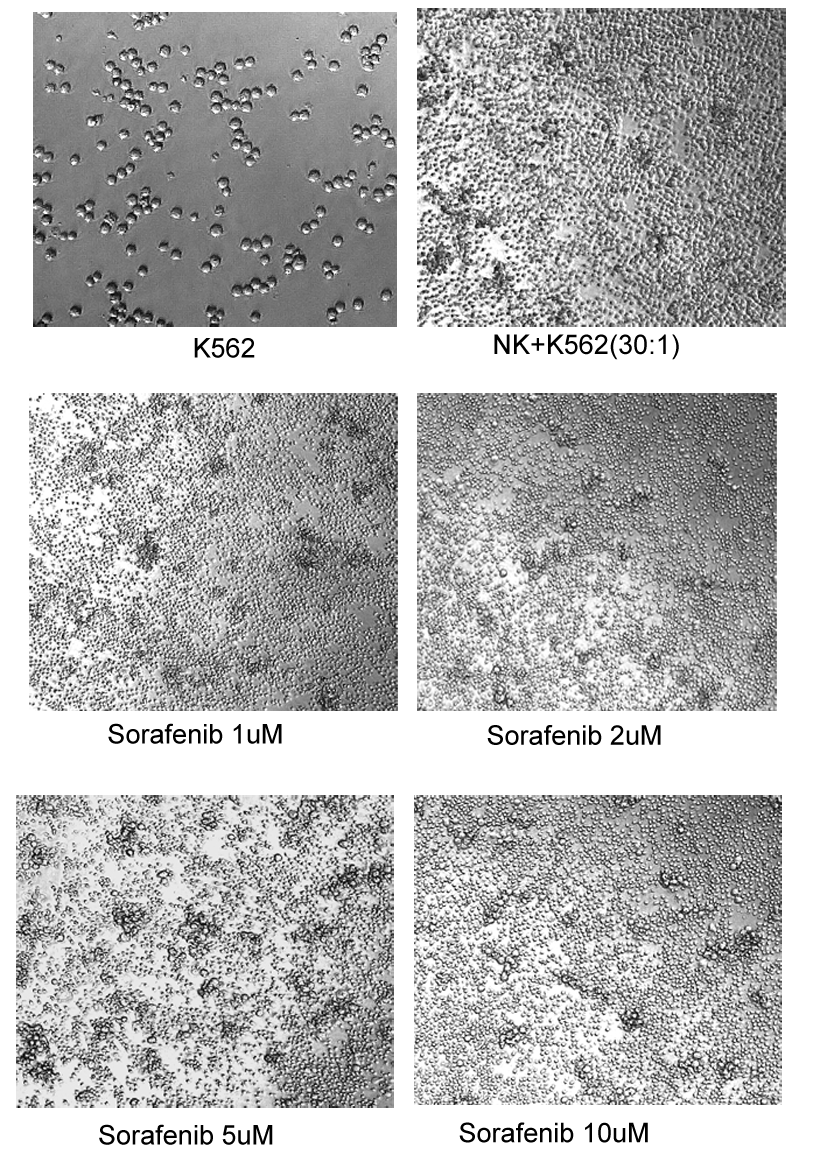

Supplement: Figure S3 — Presence of different concentrations of sorafenib reduced the lysis ratio of NK cells in response to K562 cells (E: T = 30∶1). (TIF) [file pone.0055945.s003.tif]
